# Analysis of Electromagnetic Information Leakage Based on Cryptographic Integrated Circuits

**DOI:** 10.3390/e23111508

**Published:** 2021-11-13

**Authors:** Shaofei Sun, Hongxin Zhang, Xiaotong Cui, Qiang Li, Liang Dong, Xing Fang

**Affiliations:** 1 School of Electronic Engineering, Beijing University of Posts and Telecommunications, Beijing 100876, China; sfsun@bupt.edu.cn (S.S.); cuixiaotong@bupt.edu.cn (X.C.); qiang_lee@126.com (Q.L.); dongliang@163.com (L.D.); fancy_t@bupt.edu.cn (X.F.); 2 Communication and Electronic Engineering Institute, Qiqihar University, Qiqihar 161006, China

**Keywords:** electromagnetic leakage, integrated circuits, multiple linear regression, guess entropy, electromagnetic side-channel analysis

## Abstract

Cryptographic algorithm is the most commonly used method of information security protection for many devices. The secret key of cryptographic algorithm is usually stored in these devices’ registers. In this paper, we propose an electromagnetic information leakage model to investigate the relationship between the electromagnetic leakage signal and the secret key. The registers are considered as electric dipole models to illustrate the source of the electromagnetic leakage. The equivalent circuit of the magnetic field probe is developed to bridge the output voltage and the electromagnetic leakage signal. Combining them, the electromagnetic information leakage model’s function relationship can be established. Besides, an electromagnetic leakage model based on multiple linear regression is proposed to recover the secret key and the model’s effectiveness is evaluated by guess entropy. Near field tests are conducted in an unshielded ordinary indoor environment to investigate the electromagnetic side-channel information leakage. The experiment result shows the correctness of the proposed electromagnetic leakage model and it can be used to recover the secret key of the cryptographic algorithm.

## 1. Introduction

With the rapid development of the internet of things (IoT), a large number of low cost integrated circuits have been embraced in a wide range of applications including micro-machines. Information security issues in these applications have increasingly attracted people’s attention. The emergence of side-channel analysis [1] has led to a great threat to integrated circuits. The internal information of embedded artificial intelligence devices is revealed by side-channel analysis in [2,3]. Power side-channel information is used to recover the secret key in [4]. Sensitive user information can be recovered from the acoustic side-channel of keyboard in [5]. Numerous types of side-channels have been successfully exploited to reveal secret information in previous years, such as time [6], power consumption [7], electromagnetic emission [8,9], optical signal [10] or acoustic emanation [11].

Electromagnetic side-channel analysis exploits unintentional electromagnetic leakages captured from integrated circuits to reveal secret information, especially in the area of crypto devices. Electromagnetic emission is used to identify the devices and operations in [12,13]. Screen contents are detected via electromagnetic side-channel information in [14]. Although there are several works on side-channel leakage measurements and modeling for hardware security in IoT devices [14,15], they do not consider the cryptographic algorithms. It is well known that electromagnetic side-channel analysis plays an important role in the area of crypto devices. A malicious attacker can deduce the secret key of the crypto device by analyzing the correlation between the electromagnetic leakage information and the internal states of the cryptographic algorithm. Cross devices are used to recover the secret key by electromagnetic emission in [16,17]. Near field electromagnetic side-channel analysis gains more and more attractions in recent years. Practical real-world near field electromagnetic analysis on commercial contactless smartcards in a black-box scenario is shown in [18]. The secret key is recovered from realistic cryptographic algorithm implementations using electromagnetic leakage traces in [19]. Electromagnetic side-channel analysis on PC implementations of elliptic curve cryptology is demonstrated by measuring the PC’s electromagnetic emanations in [20]. An end-to-end electromagnetic side-channel analysis system is introduced in [21]. The system combines an electromagnetic leakage scanning platform and analysis methods into a single system, which can carry out all steps automatically. Most of the previous publications mainly focus on the key recovery of cryptographic algorithms, including our previous works [22,23]. As far as we know, there are seldom works on the electromagnetic leakage model of cryptographic integrated circuits to reveal the relationship between the electromagnetic leakage signal and the cryptographic algorithm. Several works [24,25,26] just show a simple analysis about the electromagnetic leakage.

Based on previous works, we focus on the electromagnetic information leakage model of the registers related to the secret key during the process of encryption in this paper. The main contributions of the paper are as follows:

- An electromagnetic information leakage model in the process of hardware implementation is proposed to explain the relationship between the electromagnetic leakage and the secret key in detail. The registers are considered as electric dipole models to illustrate the source of the electromagnetic leakage. The equivalent circuit of the magnetic field probe is developed to bridge the output voltage and the electromagnetic leakage signal.
- An electromagnetic information leakage model based on multiple linear regression is proposed to recover the secret key of the cryptographic algorithm. The correctness of electromagnetic information leakage model also is verified by multiple linear regression according to near field tests.

The rest of this paper is organized as follows. The experimental setup is shown in Section 2. We propose the electromagnetic leakage model in Section 3. The electromagnetic leakage model based on multiple linear regression is shown in Section 4. Finally, the conclusion is presented in Section 5.

## 2. Experimental Setup

In this section, we elaborate the experimental setup in near field tests, including the devices used in the experimental platform and the cryptographic algorithm.

### 2.1. Experimental Platform

**Figure 1:**
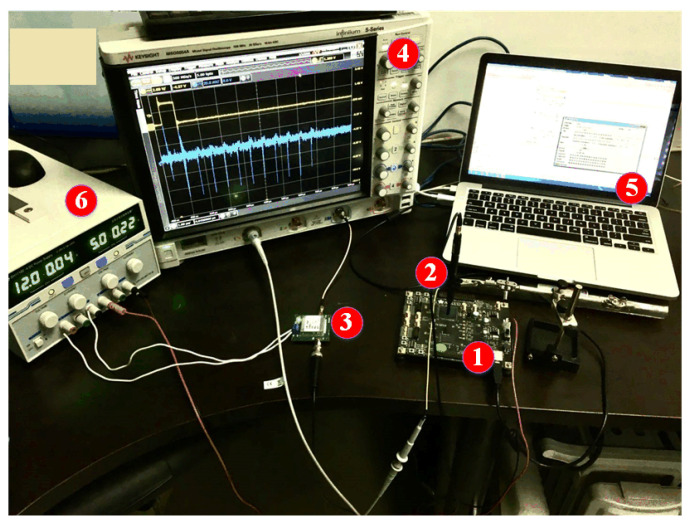
Experiment Platform.

Near field tests are conducted in an unshielded ordinary indoor environment to investigate the electromagnetic side-channel information leakage. The experiment platform is illustrated in Figure 1 which is composed of a Sakura-G board, a magnetic probe, a low noise amplifier, a Keysight oscilloscope (Keysight MSOS054A) and a direct current power supply. The Sakura-G FPGA board is used for encryption and the board is a universal test device specifically designed to standardize the security evaluation methodology of cryptographic modules on hardware security. There are two XILINX Spartan-6 chips on the board which are fabricated using 45-nm technology. One is served as the main security chip (XC6SLX75-2CSG484C), the other is the controller chip (XC6SLX9-2CSG225C). The main chip is responsible for performing the cryptographic operations and the controller chip provides the main chip with digital stimuli and controls its conditions of cryptographic operation. The board is powered by a direct current power supply in order to reduce unnecessary noise influence. A passive magnetic probe (Langer LF-B 3) is used to detect electromagnetic source of cryptographic operations. Detailed information about the probe is described in Section 3.

The main process of the experiment is shown in the following:

(1) The computer sends 128-bit plaintext and 128-bit secret key to the Sakura-G board.
(2) The Sakura-G board is responsible for encryption and returns the ciphertext to the computer.
(3) The electromagnetic leakage signal is detected by the magnetic probe and then it is amplified by the low noise amplifier.
(4) The electromagnetic leakage trace is measured by the oscilloscope and transferred to the control computer.
(5) The computer is used to receive electromagnetic leakage traces and responsible for data storage, communication and further analysis.
(6) This process is repeated many times until the electromagnetic leakage trace requirement is met.

### 2.2. Cryptographic Algorithm

**Figure 2:**
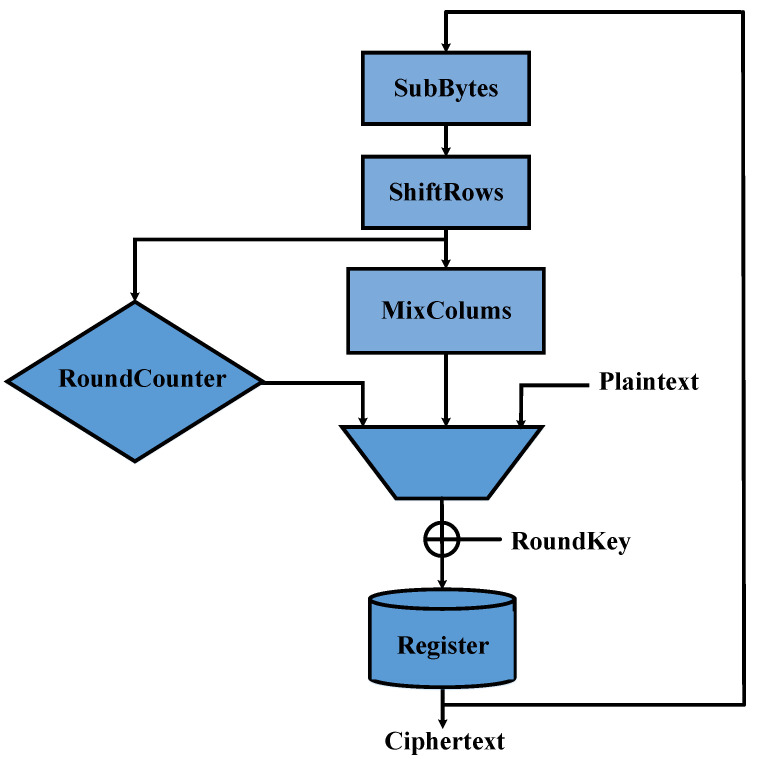
Hardware Implementation of AES.

The hardware implementation is shown in Figure 2. The benchmark of cryptographic circuit is Advanced Encryption Standard (AES) which is critical for securing many applications. AES is a symmetrical block cipher that converts 128-bit plaintexts into ciphertexts using an original 128-bit key. AES-128 algorithm has 10 rounds and every round uses a different 128-bit round key which is calculated from the original 128-bit key. There are four sub-processes per round: SubBytes, ShiftRows, MixColumns, AddRoundKey. The last round skips the MixColumns sub-processes.

According to Figure 2, the process of hardware implementation can be summarized. Firstly, the 128-bit plaintexts and 128-bit secret key are delivered by the controller chip to the main chip. The plaintexts are XORed with the original key and the output intermediate values are stored into the registers. Then the main chip performs one AES round including SubBytes, ShiftRows, MixColumns and AddRoundKey in every clock cycle and the output intermediate values after AddRoundKey are also stored into the registers. Finally, the output ciphertexts are stored into the registers after last round.

**Figure 3:**
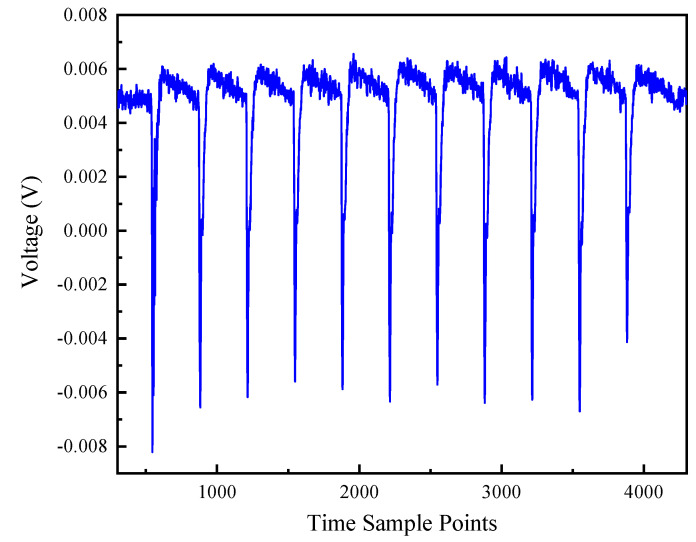
One of the measured electromagnetic trace.

One of the measured electromagnetic trace is shown in the Figure 3. We can see that there are 11 peaks in the electromagnetic trace. The first peak is the loading of plaintext into the register and the other 10 peaks are 10 rounds of the AES.

## 3. Electromagnetic Leakage Model

**Figure 4:**
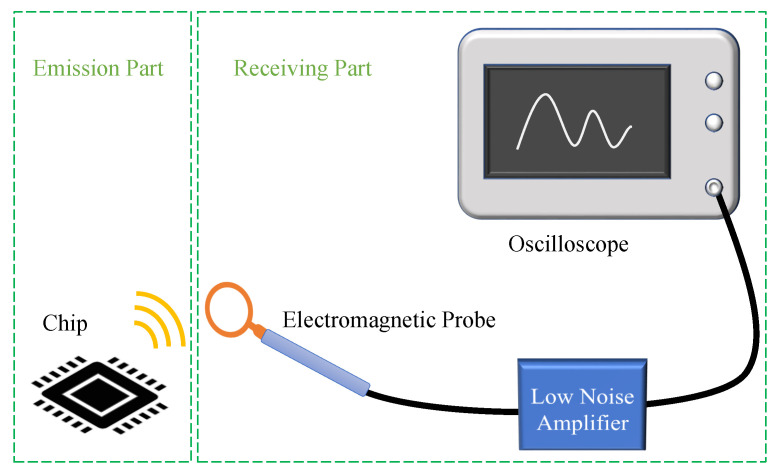
The main process of electromagnetic leakage process.

In this section, the electromagnetic leakage model will be described according to the electromagnetic leakage process. The main process can be divided into two parts: emission part and receiving part, just as shown in Figure 4. In emission part, electric dipole models are built to characterize the electromagnetic emission of the integrated circuit’s registers. In receiving part, the equivalent circuit for the magnetic probe is also given to bridge the electromagnetic leakage signal and the output voltage.

### 3.1. Source of Electromagnetic Signal Leakage

**Figure 5:**
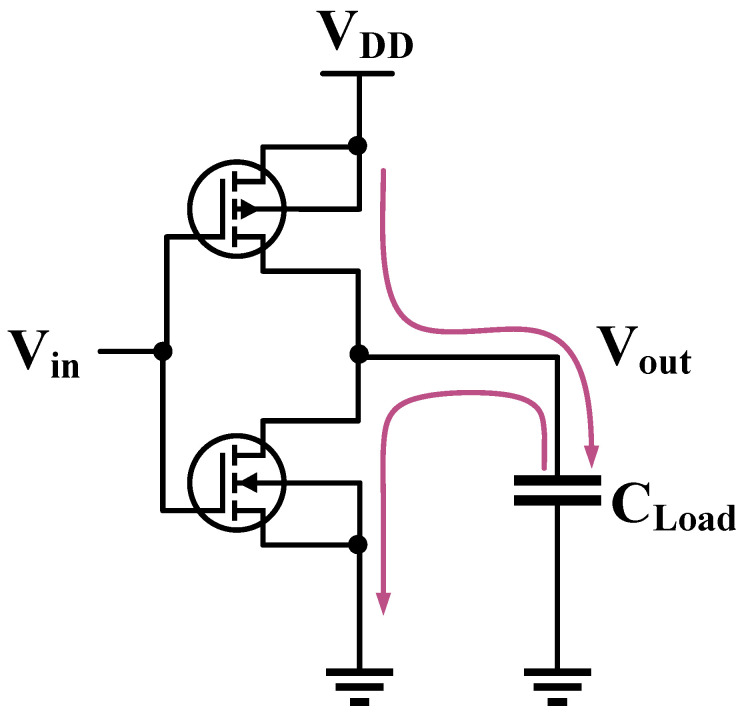
The CMOS Inverter.

Most modern integrated circuits are designed in Complementary Metal Oxide Semiconductor (CMOS) technology. It is well known that CMOS inverters are the core of almost all digital integrated circuit designs. The structure of a CMOS inverter is shown in Figure 5, which is composed of a p-channel enhanced MOS (PMOS) and a n-channel enhanced MOS (NMOS). It can be seen as a push-pull switch. Sensitive information can be leaked when the CMOS inverter is switched “ON” and “OFF”.

The electromagnetic consumption of the CMOS circuit can be divided into two parts, one is static consumption which is mainly determined by the leakage current, the other is dynamic consumption. In the static state, whether the $V_{in}$ is high (1) or low (0), the NMOS transistor and the PMOS transistor will not be turned on at the same time, one of them must be in the cut-off state, and the resistance in cut-off state is very high. So the static current is very small and the static consumption caused is also very small, accounting for about 1% of the total consumption. The static consumption is not considered in this paper due to its small proportion. Dynamic consumption is mainly switch consumption and short circuit consumption, accounting for about 99% of the total consumption. Relevant information shows that the switching consumption caused by signal inversion accounts for more than 85% of the total consumption.

When the input $V_{in}$ is low (0), the PMOS transistor is turned on and the NMOS transistor is turned off. The current flows through the PMOS transistor, the load capacitance $C_{Load}$ is charged and the external energy is consumed.

When the input $V_{in}$ is high (1), the PMOS transistor is turned off, the NMOS transistor is turned on. The current flows through the NMOS transistor, the load capacitor $C_{Load}$ is discharged and the energy is released to the outside.

In integrated circuits, information leakage is determined by the flip state of the gate circuit at the CMOS gate level. At the register level, the register is composed of multiple inverters, information leakage depends on the frequency of the inverters flip.

Ideally, the current variation of the integrated circuit is caused by the change of the logic state of the circuit. The electromagnetic leakage of integrated circuit is caused by the current flow of controller, input/output, data processing, or chip part. Most integrated circuits generally work under the control of clock signals. In each clock cycle, the corresponding short-term logic state conversion will be completed. At the same time, there will be corresponding current variations in the data processing module due to data changes. The state transition process is often completed instantaneously, and will remain in a stable state after completion until the arrival of the next clock cycle. In each clock cycle, the state transition and corresponding current variation are caused by a few bits in the data, so we can only consider the current variation in a single clock cycle.

For a certain cryptographic chip, the intermediate value state of the secret key is often stored in the registers. We consider a register as a structure similar to a CMOS inverter. When the register state changes, there will be a change in the current, resulting in electromagnetic emission. When we use the switching characteristics of the CMOS inverter, the inversion of the register state can often be completed in a short period of time, assuming that the two states before and after the *j*-th register flips are *R* and $R^{\prime }$, respectively. Therefore, the current generated by the *j*-th register flips under the controlling of the clock can be written as

(1) $$i_{j}=\alpha_{j}\beta_{j}\left(R_{j}⨁R_{j}^{\prime }\right)$$

where $\alpha_{j}$ is the *j*-th register current conversion coefficient, $\beta_{j}$ is the *j*-th register fan-out coefficient.

### 3.2. Modeling the Registers

**Figure 6:**
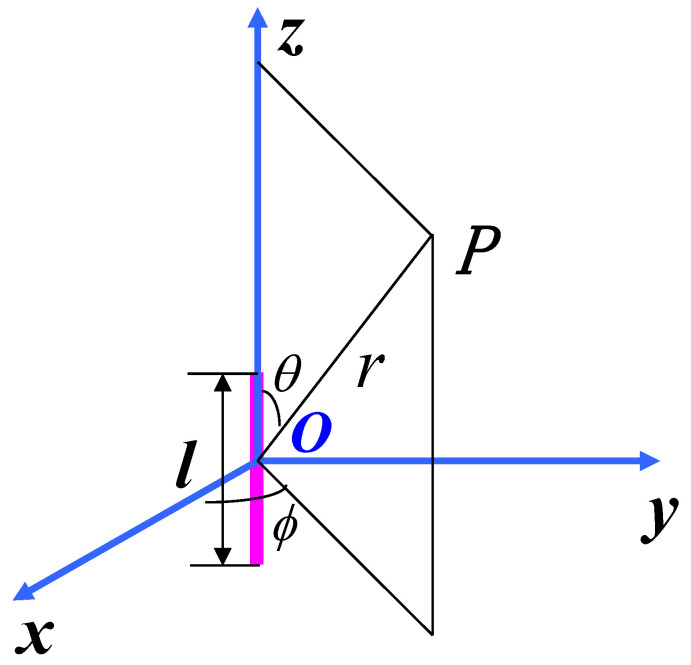
The Electric Dipole.

With the development of microelectronics technology, the manufacturing process of the chip has reached the nm level. As a result, the size of a register is also extremely small. According to the antenna theory [27], the current element is one of the most basic electromagnetic emission elements in the emitting system. Therefore, a simple and effective method is to use the current element to analyze the emission of the integrated circuit. This method is also applicable to any structure of the emitting system. The current element is usually used in electric dipoles, any actual emitting system can be decomposed into many continuous current filaments, then subdivided into electric dipoles. The field of the emitting system can be found by the sum of the contributions of these electric dipoles. Therefore, the register with current can be equivalent to an electric dipole and the current is approximately uniformly distributed. The primary electric dipole is shown in Figure 6, which is a short conducting wire of length *l*. We assume the current *I* in the short conducting wire to be uniform. The electromagnetic retarded vector potential A in Figure 6 is

(2) $$A=e_{z}\frac{\mu_{0}I\ell }{4\pi }\left(\frac{e^{-jkr}}{r}\right)$$

where $\mu_{0}$ is the permeability of free space, *r* is the distance from point *O* to point *P*, $k=\frac{2\pi }{\lambda }$.

It is convenient to use spherical coordinate system when calculating electromagnetic field, the transformation from Cartesian coordinate system to spherical coordinate system is given in Equation (3)

(3) $$\;\left[\begin{array}{c} \;A_{r}\;\\ \;A_{\theta }\;\\ \;A_{\phi }\; \end{array}\right]\;=\;\left[\begin{array}{ccc} \sin\theta \cos\phi & \sin\theta \sin\phi & \cos\theta \\ \cos\theta \cos\phi & \cos\theta \sin\phi & -\sin\theta \\ -\sin\phi & \cos\phi & 0 \end{array}\right]\;\left[\begin{array}{c} \;A_{x}\;\\ \;A_{y}\;\\ \;A_{z}\; \end{array}\right]$$

The spherical components of $A=e_{r}A_{r}+e_{\theta }A_{\theta }+e_{\phi }A_{\phi }$ are

(4) $$\begin{array}{cc} \; & A_{r}=A_{z}\cos\theta =\frac{\mu_{0}Il}{4\pi }\left(\frac{e^{-jkr}}{r}\right)\cos\theta \\ \; & A_{\theta }=-A_{z}\sin\theta =-\frac{\mu_{0}Il}{4\pi }\left(\frac{e^{-jkr}}{r}\right)\sin\theta \\ \; & A_{\phi }=0 \end{array}$$

The magnetic field strength H can be obtained

(5) $$\begin{array}{cc} H & =\frac{1}{\mu_{0}}\nabla \times A\\ \; & =-e_{\phi }\frac{Il}{4\pi }k^{2}\sin\theta \left[\frac{1}{jkr}+\frac{1}{{\left(jkr\right)}^{2}}\right]e^{-jkr} \end{array}$$

So the spherical components of the magnetic field strength H is

(6) $$\left\{\begin{array}{cc} H_{r} & =H_{\theta }=0\\ H_{\phi } & =-\frac{Il}{4\pi }k^{2}\sin\theta \left[\frac{1}{jkr}+\frac{1}{{\left(jkr\right)}^{2}}\right]e^{-jkr} \end{array}\right.$$

During the process of signal acquisition, the magnetic probe is close to the region where the cryptographic chip works. In this region, kr≪1, so this region is often called the near zone.

In the near zone, $\frac{1}{kr}\ll \frac{1}{{\left(kr\right)}^{2}}$ and $e^{-jkr}\approx 1$. The leading term in Equation (6) is

(7) $$H_{\phi }=\frac{Il}{4\pi r^{2}}\sin\theta$$

where we ignore other terms.

Therefore, the magnetic field strength H generated by the registers are

(8) $$H\left(t\right)=\sum_{j=1}^{n}\frac{l\sin\theta_{j}}{4\pi r_{j}^{2}}\alpha_{j}\beta_{j}\left(R_{j}⨁R_{j}^{\prime }\right)$$

where *n* is the number of registers.

### 3.3. Modeling the Magnetic Probe

The magnetic probe is mainly a sensing element composed of an inductive coil, which can be considered as an electromagnetic sensor. When the magnetic probe is used to detect the electromagnetic signal, the magnetic induction intensity B can pass through the receiving area S of the probe vertically by adjusting the direction of the probe. According to Faraday’s law of electromagnetic induction [28], the induced electromotive force of the probe is

(9) $$E=-N\frac{d(B\cdot S)}{dt}=-N\frac{d(\mu_{0}H\cdot S)}{dt}=-N\mu_{0}S\frac{d|H|}{dt}$$

where *N* is the number of turns of the coil in the probe.

**Figure 7:**
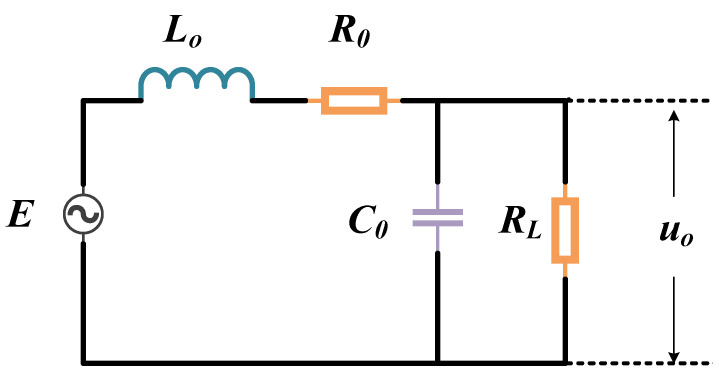
The Equivalent Circuit for Magnetic Probe.

The equivalent circuit for the magnetic probe is given in Figure 7. $L_{0}$ is the loop inductance, $C_{0}$ is the loop capacitance, $R_{L}$ is the load resistance. So the output voltage $u_{o}$ can be written as

(10) $$u_{o}=-\frac{N\mu_{0}S\cdot R_{p}\cdot \alpha_{j}\beta_{j}}{R_{L_{0}}+R_{p}}\cdot \sum_{j=1}^{n}\frac{lsin\theta_{j}}{4\pi r_{j}^{2}}\left(R_{j}⊕R_{j}^{\prime }\right)$$

where

$$R_{p}=\frac{R_{L}R_{C_{0}}}{R_{L}+R_{C_{0}}},\;R_{C_{0}}=\frac{1}{j\omega C_{0}},\;R_{L_{0}}=j\omega L_{0}$$

In the experimental environment, *l* can be regarded as constant values. For a given magnetic probe, the parameters $L_{0},C_{0},R_{L},N,S$ are constant values. Besides, the low noise amplifier provides a gain of η. Considering the noise influence, the Equation (10) can be simplified as

(11) $$\begin{array}{cc} u_{o} & =-\frac{N\mu_{0}\eta S\cdot R_{p}\cdot \alpha_{j}\beta_{j}}{R_{L_{0}}+R_{p}}\cdot \sum_{j=1}^{n}\frac{lsin\theta_{j}}{4\pi r_{j}^{2}}\left(R_{j}⊕R_{j}^{\prime }\right)+\varepsilon \\ \; & =\sum_{j=1}^{n}k_{j}\left(R_{j}⊕R_{j}^{\prime }\right)+\varepsilon \end{array}$$

where $k_{j}=-\frac{N\mu_{0}\eta S\cdot R_{p}\cdot \alpha_{j}\beta_{j}}{R_{L_{0}}+R_{p}}\cdot \frac{l\sin\theta_{j}}{4\pi r_{j}^{2}}$.

The Equation (11) is the proposed electromagnetic leakage model. We can see that the output of the magnetic probe $u_{o}$ is positively correlated with the number of flip registers $\sum_{j=1}^{n}\left(R_{j}⊕R_{j}^{\prime }\right)$.

## 4. Electromagnetic Leakage Model Based on Multiple Linear Regression

In this section, an electromagnetic leakage model based on multiple linear regression is built and the model is validated by multiple linear regression (MLR). Finally, we discuss the experimental results and recover the secret key of AES algorithm successfully.

### 4.1. Multiple Linear Regression

Multiple linear regression is usually used to study the relationship between an output variable *y* and multiple input variables denoted $X=(x_{1},\cdots ,x_{k})$.

(12) $$y=\sum_{j=0}^{k}\beta_{j}x_{j}+\varepsilon$$

where $\beta_{j}$ is the regression coefficients, $x_{0}=1$, ε is the error term.

For given *n* sets of observations, $y_{i},x_{1i},x_{2i},\cdots ,x_{ki}\;\left(i=1,2,\cdots ,n\right)$. Their relationship satisfies Equation (13).

(13) $$\left\{\begin{array}{c} y_{1}=\beta_{0}+\beta_{1}x_{11}+\cdots +\beta_{k}x_{k1}+\varepsilon_{1}\\ y_{2}=\beta_{0}+\beta_{1}x_{12}+\cdots +\beta_{k}x_{k2}+\varepsilon_{2}\\ \vdots \;\;\;\vdots \\ y_{n}=\beta_{0}+\beta_{1}x_{1n}+\cdots +\beta_{k}x_{kn}+\varepsilon_{n} \end{array}\right.$$

Multiple linear regression in matrix form is

(14) y=Xβ+ε

where

$$y=\left[\begin{array}{c} y_{1}\\ y_{2}\\ \vdots \\ y_{n} \end{array}\right],\;X=\left[\begin{array}{cccc} 1 & x_{11} & \ldots & x_{k1}\\ 1 & x_{12} & \ldots & x_{k2}\\ \vdots & \vdots & & \vdots \\ 1 & x_{1n} & \ldots & x_{kn} \end{array}\right],\;\beta =\left[\begin{array}{c} \beta_{0}\\ \beta_{1}\\ \vdots \\ \beta_{k} \end{array}\right],\;\varepsilon =\left[\begin{array}{c} \varepsilon_{1}\\ \varepsilon_{2}\\ \vdots \\ \varepsilon_{n} \end{array}\right]$$

The most popular estimation method in linear regression is least square method. The estimation of β can be calculated by

(15) $$\hat{\beta }={\left(X^{T}X\right)}^{-1}X^{T}y$$

The estimation of $\hat{y}$ is

(16) $$\hat{y}=X\hat{\beta }$$

The determination coefficient $R^{2}$ of linear estimation is a value in [0,1], which reflects the similarity of model fitting. The larger the value, the better the similarity of fitting.

(17) $$R^{2}=1-\frac{\sum_{i=1}^{n}{\left(y_{i}-{\hat{y}}_{i}\right)}^{2}}{\sum_{i=1}^{n}{\left(y_{i}-\bar{y_{i}}\right)}^{2}}$$

Comparing Equation (11) with Equation (12), if the states change of the registers $\left(R_{j}⊕R_{j}^{\prime }\right)$ is regarded as the input variable $x_{k}$, $k_{j}$ is regarded as $\beta_{k}$ and the output voltage $u_{o}$ is regarded as the output variable *y*, then the electromagnetic leakage process can be modeled by multiple linear regression. Its correctness also can be verified by the determination coefficient of multiple linear regression. The determination coefficient of the correct key should be higher than other key candidates.

The main process of electromagnetic leakage model to recover the secret key is shown in Algorithm 1.

**Algorithm 1:** Electromagnetic LeakageModel BasedOnMultiple Linear Regression.

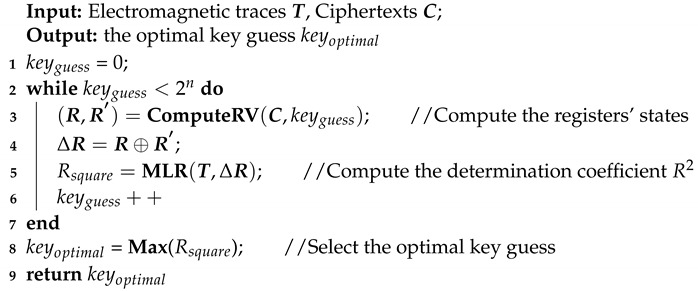



Guess entropy is a common metric used in side-channel analysis [29,30]. It is defined as the average key rank number of the correct key candidate in all key candidates with an optimal strategy. The optimal strategy means to rank the key candidates from most to least likely based on the value of the correlation analysis. The guess entropy can be used to evaluate the effectiveness of the electromagnetic leakage model. The determination coefficient can be used as the value of the correlation analysis.

### 4.2. Experiment Result

Using the experimental platform shown in Section 2, we record 6000 electromagnetic leakage traces with sampling rate 500 MSa/s. The near field experiment is conducted in an unshielded ordinary indoor environment. We use the collected electromagnetic traces to verify the electromagnetic leakage model. AES algorithm is a symmetrical block cipher algorithm, so we use single block (8 bits) as the input variables of multiple linear regression. In this paper, we implement the electromagnetic leakage model based on multiple linear regression experiments on a 2.1 GHz Intel Core E5 windows platform with 64 GB RAM.

**Figure 8:**
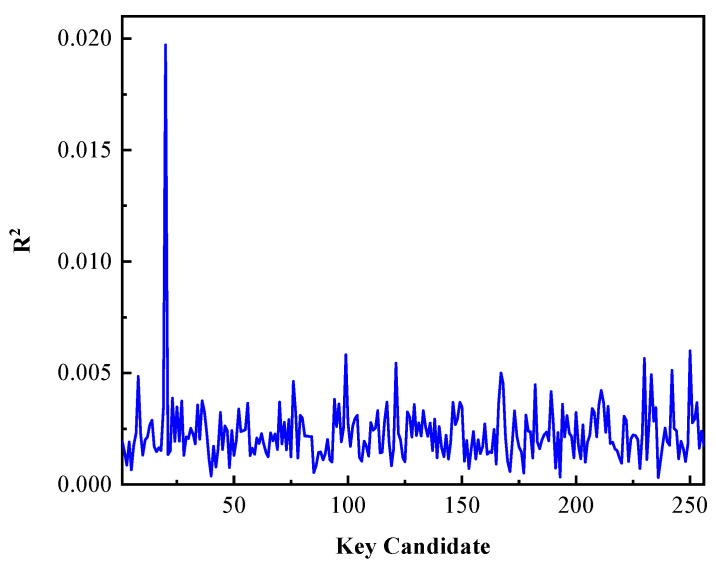
The determination coefficient of different key candidates.

**Figure 9:**
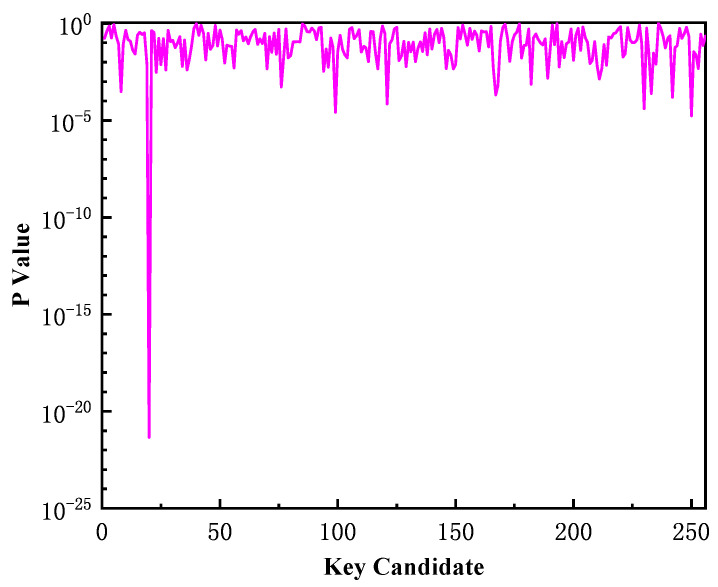
The *p* value of different key candidates.

Taking the first byte of the secret key for example, the experimental result is illustrated in Figure 8. There is an obvious higher peak at the position of 19 on the horizontal axis in Figure 8 which indicates the correct key is 19 and the fitting similarity of the correct key is much better than that of other key candidates. According to the test for overall significance, the *p* values of different key candidates are shown in Figure 9. We can see that when the key candidate is 19, its *p* value is $4.54\times 10^{-22}$. It can be considered to reject the null hypothesis.

**Figure 10:**
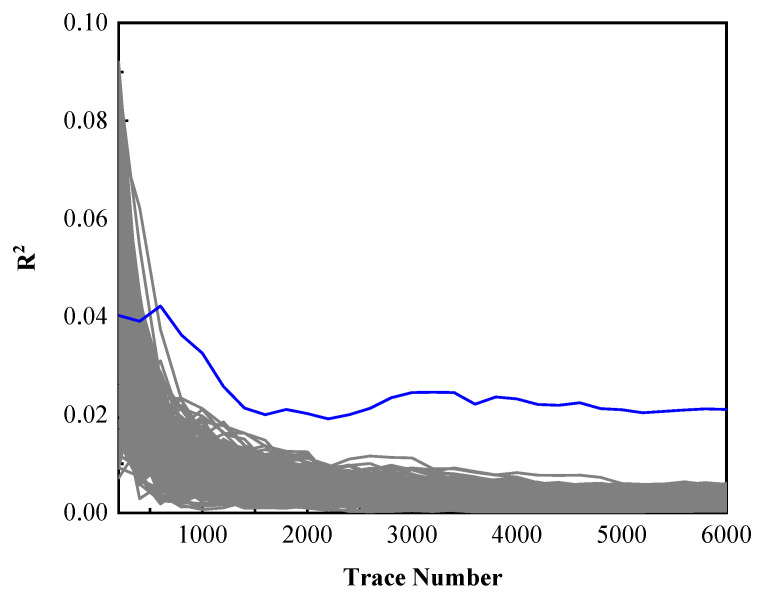
The relationship between determination coefficient and trace number.

The relationship between determination coefficient $R^{2}$ and the number of electromagnetic traces is shown in Figure 10. Blue line is the determination coefficient $R^{2}$ of the correct key, the other lines are other key candidates. The determination coefficient of the correct key is separated from the others with the increasing number of electromagnetic traces. It needs about 600 electromagnetic traces to recover the secret key successfully. When the number of trace is less than 600, it’s difficult to distinguish them because of noise influence. More electromagnetic traces can eliminate the influence of noise to a certain extent.

**Figure 11:**
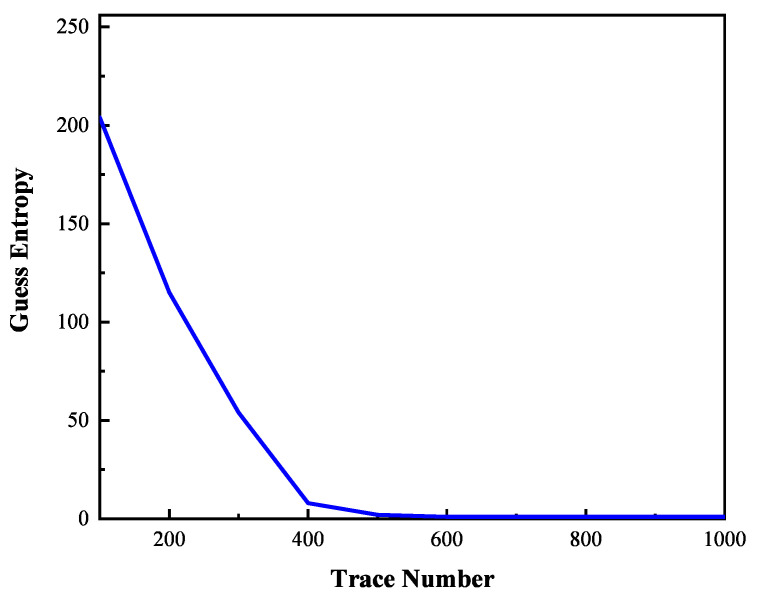
The guess entropy of the proposed model in different trace number.

In order to evaluate the effectiveness of the electromagnetic leakage model, partial guess entropy of the correct key candidate is calculated and is shown in Figure 11. According to Figure 10, it can be known that when the number of electromagnetic trace is more than 1000, the correct key candidate is already recovered. More electromagnetic traces can not provide further help except to increase the computational complexity. Therefore, we only consider the partial guess entropy within 1000. When the number of electromagnetic trace is small, the partial guess entropy is lower than 200. It is difficult to recover the key under this condition. With a smaller number of traces increase, the guessing entropy is significantly reduced. When the number of electromagnetic trace is 400, the guess entropy is ranked in the first few positions. Around 600 electromagnetic traces, the partial guess entropy falls to the first position indicating the key is recovered correctly. When the number of electromagnetic trace is more than 600, the partial guess entropy is in a stable state and remains in the first position. The partial guess entropy also proves that the proposed electromagnetic leakage model can be used to recover the key of the cryptographic algorithm.

## 5. Conclusions

In this paper, we proposed an electromagnetic information leakage model to explore the secret key in cryptographic integrated circuits. The electric dipole models were built to characterize the electromagnetic leakage. A magnetic probe was used to receive electromagnetic leakage signal and bridge the output voltage and the electromagnetic leakage signal. Both of them constituted the electromagnetic information leakage model. The model illustrated the relationship between the electromagnetic leakage signal and secret key. Besides, we proposed an electromagnetic leakage model based on multiple linear regression to recover the secret key. The correctness of the model was validated by multiple linear regression according to near field tests and its effectiveness was evaluated by guess entropy. The experiment results showed the proposed electromagnetic leakage model can be used to recover the secret key of the cryptographic algorithm.

The electromagnetic leakage model also can be applied to other micro cryptographic devices, such as smart cards, embedded devices, microcomputers, and other micro-machines, because these devices have the similar physical structure. Other cryptographic algorithms also can use a similar method to characterize the electromagnetic information leakage. However, we haven’t done the relevant experiments on these devices which remain to be studied in the future. Besides, the specific values in the electromagnetic leakage model will also be explored in our future work.

## Data Availability

Data is contained within the article.
